# Breast-Lesion Characterization using Textural Features of Quantitative Ultrasound Parametric Maps

**DOI:** 10.1038/s41598-017-13977-x

**Published:** 2017-10-20

**Authors:** Ali Sadeghi-Naini, Harini Suraweera, William Tyler Tran, Farnoosh Hadizad, Giancarlo Bruni, Rashin Fallah Rastegar, Belinda Curpen, Gregory J. Czarnota

**Affiliations:** 1 Department of Medical Biophysics, University of Toronto, Toronto, ON Canada; 2 Physical Sciences, Sunnybrook Research Institute, Sunnybrook Health Sciences Centre, Toronto, ON Canada; 3 Department of Radiation Oncology, Odette Cancer Centre, Sunnybrook Health Sciences Centre, Toronto, ON Canada; 4 Department of Radiation Oncology, University of Toronto, Toronto, ON Canada; 5 Centre for Health and Social Care Research, Sheffield Hallam University, Sheffield, UK; 6 Department of Medical Imaging, Sunnybrook Health Sciences Centre, Toronto, ON Canada

## Abstract

This study evaluated, for the first time, the efficacy of quantitative ultrasound (QUS) spectral parametric maps in conjunction with texture-analysis techniques to differentiate non-invasively benign versus malignant breast lesions. Ultrasound B-mode images and radiofrequency data were acquired from 78 patients with suspicious breast lesions. QUS spectral-analysis techniques were performed on radiofrequency data to generate parametric maps of mid-band fit, spectral slope, spectral intercept, spacing among scatterers, average scatterer diameter, and average acoustic concentration. Texture-analysis techniques were applied to determine imaging biomarkers consisting of mean, contrast, correlation, energy and homogeneity features of parametric maps. These biomarkers were utilized to classify benign versus malignant lesions with leave-one-patient-out cross-validation. Results were compared to histopathology findings from biopsy specimens and radiology reports on MR images to evaluate the accuracy of technique. Among the biomarkers investigated, one mean-value parameter and 14 textural features demonstrated statistically significant differences (p < 0.05) between the two lesion types. A hybrid biomarker developed using a stepwise feature selection method could classify the legions with a sensitivity of 96%, a specificity of 84%, and an AUC of 0.97. Findings from this study pave the way towards adapting novel QUS-based frameworks for breast cancer screening and rapid diagnosis in clinic.

## Introduction

Breast cancer still remains the most commonly diagnosed cancer type in women accounting for 27% of all incident cases annually^1^. One in eight women will develop breast cancer during her lifetime and one in 30 women will die from it^1^. Rapid and accurate diagnosis and characterization of breast cancer plays a vital role in treatment planning and improved prognosis. The current screening workflow for breast-cancer diagnosis begins with x-ray mammography, followed by standard ultrasound imaging (B-mode), dynamic contrast-enhanced magnetic-resonance imaging (DCE-MRI), and core-needle biopsy, as needed. Statistical reports indicate that patients with dense breasts have a high chance of receiving a false negative result for lesion detection based on mammography^2^. Biopsy remains the gold standard approach for pathological confirmation of malignancy and tumour grade characterization^3^. However, biopsies are invasive, painful and also carry a risk of tumour cell migration. Furthermore, many biopsies are also conducted unnecessarily due to “over-diagnosis’ as a result of the low specificity of ultrasound B-mode images^4^. DCE-MRI may improve the specificity of breast cancer diagnosis; however MRI is expensive and is often not available for rapid diagnosis due to the longer wait time associated with it compared to mammography and ultrasound. Development of inexpensive and potentially widely-available imaging techniques that can rapidly characterize breast masses with a high sensitivity and specificity is highly beneficial for the early diagnosis of breast cancer and triaging patients in screening workflows.

One limitation associated with standard ultrasound imaging for breast cancer screening is that ultrasound B-mode images are mainly qualitative and fail to provide reliable quantitative information about underlying tissue microstructure^5^. Quantitative ultrasound (QUS) techniques have been introduced to address such limitation^6^. Such techniques analyze ultrasound radiofrequency (RF) raw data, before it is envelope detected, log amplified, and processed to form B-mode ultrasound images. These methods extract quantitative measures describing intrinsic acoustic characteristics of tissue related to underlying micro-structures^7,8^. Effects of operator and instrument-setting variables are often minimized in such methods by normalizing ultrasound signals against a reference in a frequency-dependent manner^9,10^. A number of QUS spectral parameters including mid-band fit (MBF), spectral slope (SS) and spectral intercept (SI) are derived by linear regression analysis on the normalized power spectrum of ultrasound RF data^10,11^. These are the parameters that can be linked to scattering power and the size and concentration of acoustic scatterers^12,13^. Two other parameters, effective scatterer diameter (ESD) and effective acoustic concentration (EAC), are derived by fitting a form-factor model to the backscatter coefficient (BSC) that can be estimated using the normalized power spectrum^14^. The EAC parameter is defined as the product of the average number of scatterers per unit volume and the relative acoustic-impedance difference between the scatterers with the surrounding tissue^15,16^. Another QUS parameter, the spacing among scatterers (SAS), represents the distance between the coherent periodically-arranged scatters within tissue, and can be estimated by computing the autocorrelation of the normalized power spectrum estimated by an autoregressive (AR) model^17^.

Techniques using QUS have been demonstrated to be capable of detecting tumour response to treatment in preclinical models^10,18^ and in clinical settings^11,16,19–22^, differentiating between various tissue types in prostate, liver, and retina^23–29^, determining blood-clot and intravascular-plaque compositions^30–32^, and detecting the presence of tumour deposits in lymph nodes *ex vivo* ^33^. A study on preclinical animal tumour models demonstrated that QUS techniques have the ability to differentiate between normal and cancerous thyroid tissues. In particular, parameters quantifying the size and concentration of acoustic scatterers could be used to classify normal thyroid tissue and C-cell adenoma (benign) versus papillary thyroid carcinoma (PTC) and follicular variant papillary thyroid carcinoma (FV-PTC)^34^. With regards to the breast cancer, previous preclinical studies have demonstrated that QUS parameters are able to differentiate between spontaneously occurring mammary fibroadenomas (benign) and mammary carcinomas (malignant), and also differentiate between two types of mammary cancers: carcinoma and sarcoma^35,36^. Another study demonstrated that glandular acini are the most prevalent source of scattering in the fibroadenomas. Therefore, QUS parametric images indicative of the size of acoustic scatterers could potentially provide a good distinction between benign versus malignant lesions^37^. QUS techniques have also demonstrated the capability to differentiate breast tumours from the surrounding normal tissue in locally advanced disease^3^.

Heterogeneity in tumour micro-structure, physiology and metabolism has demonstrated diagnostic and prognostic values in cancer characterization^38–44^. Spatial heterogeneity in various characteristics of tumour can be detected in images acquired with different modalities including MRI^45,46^, positron emission tomography (PET)^47,48^, computed tomography (CT)^49,50^, and diffuse optical spectroscopy (DOS)^51^. Such heterogeneity can be quantified using texture-analysis techniques^52^. Texture-analysis techniques have been applied to ultrasound B-mode images in order to quantify spatial heterogeneities within tumour in tissue characterization applications, such as discriminating between benign and malignant breast tumours^53–55^. The principle behind this approach is that benign and malignant lesions often demonstrate homogeneous and heterogeneous internal echoes, respectively. Texture-analysis techniques can quantify the spatial alterations in internal echo properties of tissue based on the ultrasonic gray-level transitions, and hence can define differentiable characteristics in this application. However, conventional ultrasound B-mode images may present undesirable variations in estimates of texture due to variations in instruments settings, ultrasound beam diffraction, and attenuation effects. Such limitations can be addressed by performing texture analysis on QUS parametric images in which these artifacts have been compensated. In this context, a recent study has reported significant differences in textural features determined from QUS parametric images at pre-treatment between responding and non-responding breast cancer patients to neo-adjuvant chemotherapy^56^. Specifically, the significant parameters were measures of inhomogeneity in size and concentration of scatterers within tumours. Another study recently demonstrated that QUS textural parameters can characterize tumours in terms of histological grade^15^.

The study described here has investigated the efficacy of QUS spectral parametric imaging in conjunction with texture-analysis techniques to differentiate benign versus malignant breast lesions using data acquired from 78 patients with suspicious breast lesions. QUS spectral analysis techniques were performed on ultrasound RF data to generate parametric maps of MBF, SS, SI, SAS, ESD, and EAC. Several textural features were determined from each parametric map in addition to an average-based mean-value parameter as imaging biomarkers. These biomarkers were utilized to classify benign versus malignant lesions using a k-nearest neighbour (K-NN) classifier with leave-one-patient-out cross-validation. Results were compared to findings from biopsy specimens and magnetic resonance (MR) images to evaluate performance of the technique. Among the QUS parameters investigated, one mean-value parameter and 14 textural features demonstrated statistically significant differences between the benign versus malignant lesions. An accuracy of greater than 80% was achieved by eight single biomarkers used to classify the two lesion types. A hybrid biomarker developed using a stepwise feature selection method classified the benign and malignant breast legions with an accuracy of 91% and an area under the receiver operating characteristic (ROC) curve (AUC) of 0.97. The observations in this study suggest that QUS spectral parametric imaging along with texture-analysis methods have a high potential to characterize suspicious breast lesions rapidly, non-invasively, and with high sensitivity and specificity.

## Material and Methods

### Study Protocol and Data Acquisition

The study was conducted in accordance with institutional research-ethics-board approval (Sunnybrook Health Sciences Centre). After obtaining informed consent, ultrasound B-mode images and radiofrequency (RF) data were acquired from 78 patients recruited in the Rapid Diagnostic Unit (RDU) of Louise Temerty Breast Cancer Centre at Sunnybrook Health Sciences Centre, Toronto, Ontario, Canada. Data acquisition was performed by an experienced sonographer using a SonixTouch system (Ultrasonix, Vancouver, Canada) equipped with a linear array transducer (L14-5/60 W) with a centre frequency of ~6 MHz and sampling rate of 40 MHz. Data were acquired along 512 lateral scan lines (6 cm lateral field of view) with an imaging depth of 4 cm. The focus of transducer was set at the midline of the tumour. Ultrasound images and RF data were acquired at about 5 mm intervals over the entire tumour volume. Dynamic contrast-enhanced MR images and core biopsy specimens were acquired from the patients as gold standard approaches for breast-lesion characterization and cancer diagnosis. Clinical reports including results from MR images and biopsy specimens were used as ground truth to identify benign versus malignant lesions.

### Feature Extraction and Data Analysis

QUS analysis was performed over a region-of-interest (ROI) covering the whole lesion in each imaging plane. Spatial parametric images were generated for each QUS parameter applying a sliding window analysis technique with a 2 mm by 2 mm sliding window (containing 17 scan lines with 102 samples in each line) and a 94% (1.88 mm) overlap between adjacent windows in axial and lateral directions. A Hanning apodization was applied on individual scan lines within the sliding window. The size of the sliding window was selected to cover enough number of ultrasound wavelengths for reliable spectral analysis while preserving texture in parametric images. The overlap between adjacent windows was selected to obtain parametric images with isotropic pixels (0.12 mm × 0.12 mm). A total of six QUS parameters were investigated (described below) including MBF, SS, SI, SAS, ESD, and EAC.

The mean normalized power spectrum was computed for each window via fast Fourier transform and phantom data normalization. Normalization was performed to remove the effects of system transfer functions and transducer beam-forming using reference data obtained with the same scan settings from a tissue-mimicking phantom. The reference phantom was comprised of 5–30 µm glass beads embedded in a homogenous medium of microscopic oil droplets that were sunk in gelatin, and had an attenuation coefficient of 0.576 dB/MHz/cm and a speed of sound at 1488 m/s (University of Wisconsin, Department of Medical Physics, Madison, WI, USA). Attenuation correction and linear regression analysis were performed on mean normalized power spectrum and spectral parameters including the MBF, SS, SI were derived^57^. A two-layer (intervening tissue and tumour) attenuation correction was performed using total attenuation estimation^58^. The attenuation coefficient estimate (ACE) of tumour was calculated using a spectral difference method by estimating the rate of change in the spectral power magnitude with depth (over the tumour region) and frequency relative to the reference phantom^59^. An attenuation coefficient of 1 dB/MHz.cm was assumed for intervening breast tissue based on ultrasound tomography measurements of the breast^60^. Attenuation correction was performed using the point-compensation method^61^. The ESD and EAC parameters were derived by fitting a spherical Gaussian form-factor model to the BSC estimated using attenuation-corrected normalized power spectrum^14^.

To derive the SAS parameter, the power spectrum of sample was estimated using an autoregressive (AR) model and the AR model parameters were estimated using Burg’s recursive algorithm^62^. The power spectrum was then normalized to that of a planar reflector. The planar reflector normalization was performed at different depths using pre-recorded reference RF data acquired from a Plexiglas-water interface at different depths. For each RF block in the sample image, a reference RF block was selected using the nearest neighbour approach. By computing the autocorrelation of the normalized power spectrum, the SAS parameter was determined from the frequency at which the peak occurred in the autocorrelation. The method used here for SAS estimation is described in detail in ref.^3^.

In addition to an average-based mean-value parameter derived, textural parameters were extracted from each QUS parametric map using the method of gray-level co-occurrence matrix (GLCM)^52^. The GLCM represents the spatial relationship between the neighbouring pixels within an image. The full range of gray-level intensities in each parametric image was linearly scaled and quantized into 16 levels. Symmetric GLCMs were calculated for each parametric image at five inter-pixel distances (1, 2, 3, 4 and 5 pixels) and at four directions (0°, 45°, 90° and 135°). Four textural features including contrast, correlation, energy and homogeneity were extracted from each GLCM using the equations 1–4, respectively, and were subsequently averaged over all GLCMs of each parametric image.

1 $$Contrast=\sum _{|i-j|=0}^{{N}_{g}-1}|i-j{|}^{2}\sum _{i=1}^{{N}_{g}}\sum _{j=1}^{{N}_{g}}p(i,j)$$

2 $$Correlation=\frac{{\sum }_{i=1}^{{N}_{g}}{\sum }_{j=1}^{{N}_{g}}(i-{\mu }_{i})(j-{\mu }_{j})p(i,j)}{{\sigma }_{i}{\sigma }_{j}}$$

3 $$Energy=\sum _{i=1}^{{N}_{g}}\sum _{j=1}^{{N}_{g}}p{(i,j)}^{2}$$

4 $$Homogeneity=\sum _{i=1}^{{N}_{g}}\sum _{j=1}^{{N}_{g}}\frac{p(i,j)}{1+\,|i-j|}$$

In equations 1–4, p(i, j) is the probability of having two neighbour pixels with gray-level intensities of i and j in the map, and N_g_ is the number of quantized gray-level intensities. The *μ* and *σ* are the mean and standard deviations for row *i* or column *j* of the GLCM matrix. The contrast parameter quantifies local gray-level variation of an image, the correlation parameter represents the linear dependency among neighbouring pixels, the energy parameter measures textural uniformity within neighbouring pixels, and the homogeneity parameter quantifies the incidence of pixel pairs of different intensities.

### Statistical Data Analysis

Each QUS mean-value and textural parameter was calculated for all scan planes and subsequently averaged across the entire volume of tumour. Statistical analysis was performed using non-parametric Mann-Whitney U test (two-sided, 95% confidence) to assess for any statistically significant differences between benign versus malignant lesions using the QUS biomarkers (PASW Statistics 18, SPSS Inc., Chicago, IL). A stepwise linear discriminant analysis (LDA) was carried out to determine the best combination of parameters that significantly contributes to a hybrid biomarker to separate the two lesion types linearly. A K-NN classification was used to evaluate the efficacy of QUS parameters to differentiate benign versus malignant lesions non-invasively^63^. Cross-validated sensitivity, specificity and accuracy were calculated, in addition to the AUC, to measure the performance of the classification. The leave-one-patient-out method was applied for cross-validation of classification.

### Data Availability Statement

Data were collected and available at the Odette Cancer Centre, Sunnybrook Health Sciences Centre, Toronto, ON, Canada.

## Results

**Figure 1:**
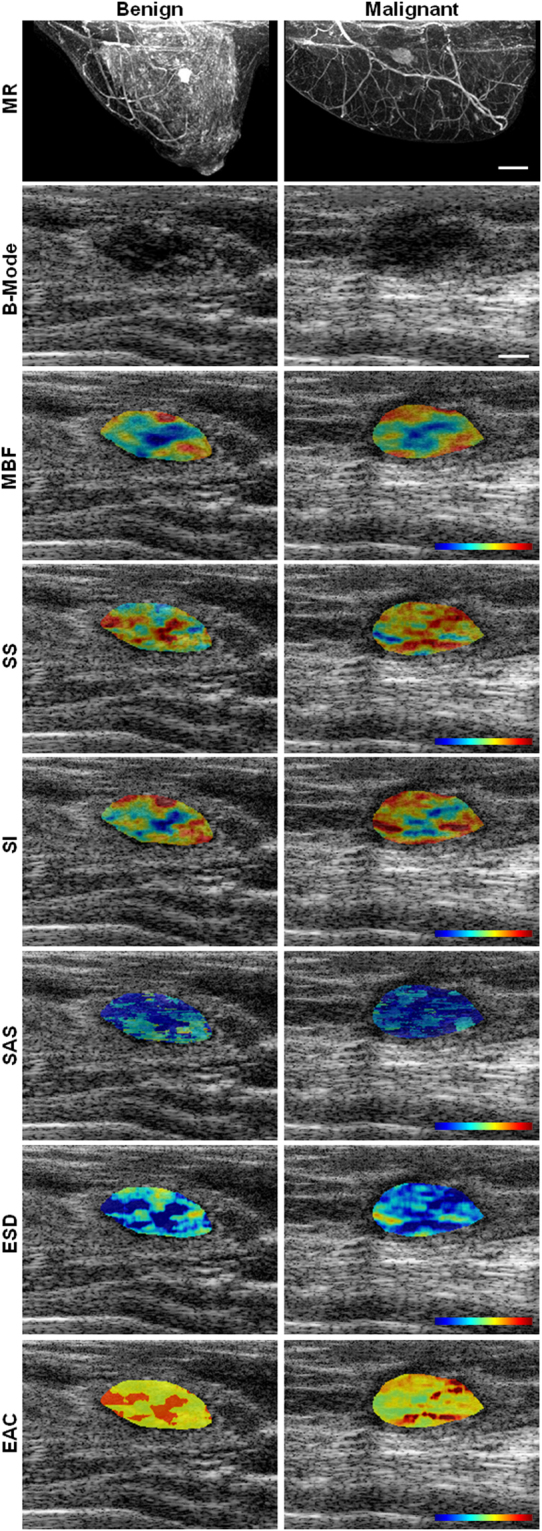
Representative MR and ultrasound B-mode images with QUS parametric overlays of MBF, SS, SI, SAS, ESD, EAC obtained from a benign and a malignant lesion. The color bar represents a scale encompassing 20 dBr for MBF, 5 dBr/MHz for SS, 30 dBr for SI, 3 mm for SAS, 160 µm for ESD, and 50 dB/cm^3^ for EAC. The scale bar represents 2 cm and 5 mm in MR and ultrasound images, respectively.

Among the 78 patients that participated in this study, 46 and 32 patients were confirmed with benign and malignant lesions, respectively, based on radiology and pathology reports. The patients had an average age of 52 ± 14 years, and an average tumour size of 2.2 ± 1.7 cm with respect to the largest lesion dimension. Representative MR and ultrasound images obtained from clinically-confirmed benign and malignant masses are presented in Fig. 1. Ultrasound B-mode images are demonstrated along with parametric overlays of MBF, SS, SI, SAS, ESD and EAC features. Overall, mean-value and textural parameters determined from these parametric maps were used to form quantitative “fingerprints” associated with benign versus malignant lesions.

**Figure 2:**
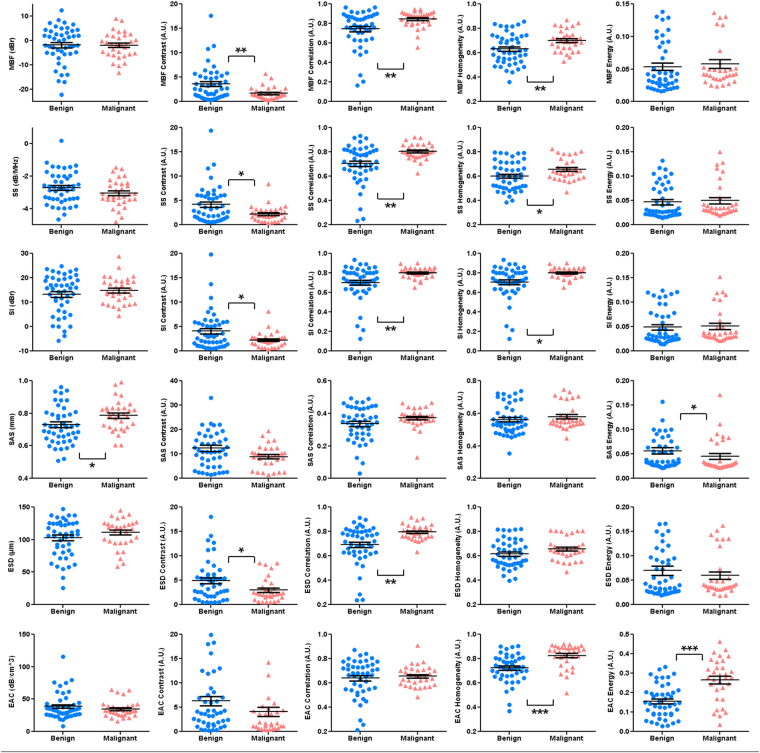
Scatter plots of the QUS mean-value and textural parameters for benign versus malignant lesions. Statistically significant (p < 0.5), highly significant (p < 0.01), and extremely significant (p < 0.001) differences are shown with *^,^ **, and ***, respectively.

Figure 2 presents scatter plots associated with the mean-value and textural parameters derived from each of the QUS parametric images for the two lesion types. The MBF-derived mean, contrast, correlation, energy and homogeneity parameters demonstrated average values of −1.8 ± 1.1 dBr versus −1.9 ± 0.9 dBr, 3.6 ± 0.5 A.U. versus 1.7 ± 0.2 A.U., 0.7 ± 0.03 A.U. versus 0.9 ± 0.01 A.U., 0.6 ± 0.02 A.U. versus 0.7 ± 0.02 A.U., and 0.05 ± 0.01 A.U. versus 0.06 ± 0.01 A.U. for benign versus malignant lesions, respectively. The mean-value and textural parameters determined from the SS parametric maps demonstrated average values of −2.7 ± 0.1 dB/MHz versus −3.0 ± 0.1 dB/MHz, 4.2 ± 0.6 A.U. versus 2.2 ± 0.3 A.U., 0.7 ± 0.02 A.U. versus 0.8 ± 0.01 A.U., 0.6 ± 0.02 A.U. versus 0.7 ± 0.02 A.U., and 0.05 ± 0.01 A.U. versus 0.05 ± 0.01 A.U. for the two lesion types, respectively. The parameters obtained from the SI parametric maps showed average values of 13.2 ± 1.2 dBr versus 14.9 ± 0.9 dBr, 4.1 ± 0.6 A.U. versus 2.2 ± 0.3 A.U., 0.7 ± 0.03 A.U. versus 0.8 ± 0.01 A.U., 0.6 ± 0.02 A.U. versus 0.7 ± 0.01 A.U., and 0.05 ± 0.01 A.U. versus 0.05 ± 0.01 A.U. for these lesion types, respectively. The SAS mean, contrast, correlation, energy and homogeneity parameters demonstrated average values of 0.7 ± 0.02 mm versus 0.8 ± 0.02 mm, 12.3 ± 1.4 A.U. versus 9.0 ± 0.8 A.U., 0.3 ± 0.02 A.U. versus 0.4 ± 0.01 A.U., 0.6 ± 0.01 A.U. versus 0.6 ± 0.01 A.U., and 0.06 ± 0.01 A.U. versus 0.04 ± 0.01 A.U. for benign versus malignant lesions, respectively. The mean-value and textural parameters extracted from the ESD parametric maps demonstrated average values of 102.6 ± 4.4 µm versus 111.1 ± 4.1 µm, 4.9 ± 0.6 A.U. versus 3.0 ± 0.4 A.U., 0.7 ± 0.02 A.U. versus 0.8 ± 0.01 A.U., 0.61 ± 0.02 A.U. versus 0.66 ± 0.02 A.U., and 0.07 ± 0.01 A.U. versus 0.06 ± 0.01 A.U. for the two lesion types, respectively. The parameters obtained from the EAC parametric maps showed average values of 38.8 ± 2.9 dB/cm^3^ versus 34.5 ± 2.2 dB/cm^3^, 6.3 ± 1.0 A.U. versus 4.1 ± 0.9 A.U., 0.6 ± 0.02 A.U. versus 0.7 ± 0.01 A.U., 0.7 ± 0.02 A.U. versus 0.8 ± 0.02 A.U., and 0.2 ± 0.01 A.U. versus 0.3 ± 0.02 A.U. for these lesion types, respectively. The benign and malignant lesions demonstrated ACE mean-values of 1.3 ± 0.2 dB/MHz.cm and 0.8 ± 0.1 dB/MHz.cm, respectively.

Among the mean-value parameters, only SAS demonstrated a statistically significant difference between the benign and malignant lesions (p = 0.016). However, several QUS textural parameters including MBF contrast (p = 0.008), correlation (p = 0.004) and homogeneity (p = 0.009), SS contrast (p = 0.027), correlation (p = 0.002) and homogeneity (p = 0.027), SI contrast (p = 0.032), correlation (p = 0.001) and homogeneity (p = 0.034), SAS energy (p = 0.049), ESD contrast (p = 0.035) and correlation (p = 0.002), as well as EAC homogeneity (p < 0.001) and energy (p < 0.001) exhibited significant differences between the two lesion types.

**Figure 3:**
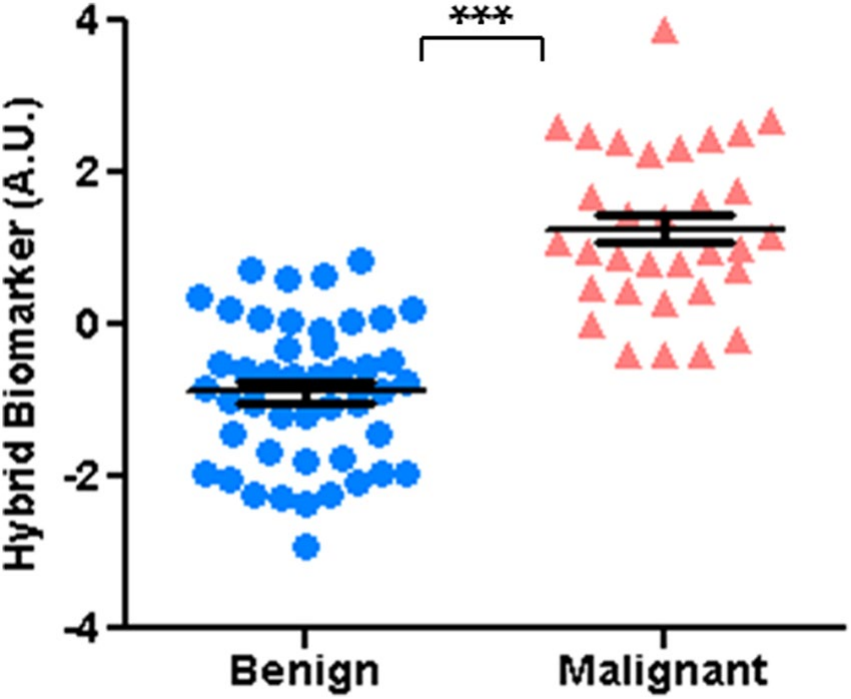
Scatter plot of the hybrid QUS biomarker for benign versus malignant lesions, demonstrating a statistically extremely significant difference between the lesion types (p < 0.001).

Table 1 summarizes result of the stepwise linear discriminant analysis performed to form a hybrid QUS biomarker for lesion characterization. Seven parameters (out of 30) demonstrated a significant contribution to the model and were incorporated based on their level of contribution with the standardized coefficients presented in Table 1. Specifically, EAC homogeneity, EAC energy, MBF homogeneity, SS correlation, SAS homogeneity, MBF and ESD energy demonstrated the highest to lowest contributions towards the model. Figure 3 demonstrates a scatter plot of the hybrid QUS biomarker for the benign versus malignant lesions. A very good separation was provided by the hybrid biomarker between the two lesion types that was found to be statistically extremely significant (p < 0.001).

**Table 1:** The parameters with significant contribution to the hybrid QUS biomarker, identified through a stepwise linear discriminant analysis.

Parameter	Standardized Coefficient
EAC Homogeneity	−2.30
EAC Energy	2.21
MBF Homogeneity	1.42
SS Correlation	1.26
SAS Homogeneity	−0.79
MBF	0.77
ESD Energy	−0.73

Table 2 presents results of the cross-validated classification of breast lesions using the K-NN classifier. The results are presented for each single QUS parameter as well as the hybrid QUS feature vector. The hybrid feature vector was composed of the seven QUS parameters identified earlier through the stepwise approach. An accuracy of over 80% was achieved by a number of single QUS parameters, including MBF energy (sensitivity = 87%, specificity = 72%, AUC = 0.93), SI homogeneity (sensitivity = 83%, specificity = 81%, AUC = 0.93), SAS (sensitivity = 87%, specificity = 78%, AUC = 0.95), SAS correlation (sensitivity = 87%, specificity = 78%, AUC = 0.93), ESD homogeneity (sensitivity = 87%, specificity = 72%, AUC = 0.90), EAC (sensitivity = 83%, specificity = 78%, AUC = 0.90), EAC homogeneity (sensitivity = 93%, specificity = 66%, AUC = 0.94), and EAC energy (sensitivity = 85%, specificity = 78%, AUC = 0.94). The hybrid QUS feature vector could classify the benign and malignant breast legions with a sensitivity, specificity, and accuracy of 96%, 84% and 91%, respectively, and an AUC of 0.97.

**Table 2:** Cross-validated results of breast-lesion classification using different QUS biomarkers.

Parameter	Sensitivity (%)	Specificity (%)	Accuracy (%)	AUC
MBF	78.3	68.7	74.4	0.86
MBF Contrast	84.8	59.4	74.4	0.87
MBF Correlation	82.6	71.9	78.2	0.91
MBF Homogeneity	78.2	75.0	76.9	0.92
MBF Energy	87.0	71.9	80.8	0.93
SS	78.3	62.5	71.8	0.86
SS Contrast	80.4	53.1	69.2	0.84
SS Correlation	78.3	68.7	74.4	0.86
SS Homogeneity	82.6	56.3	71.8	0.85
SS Energy	84.8	68.8	78.2	0.90
SI	87.0	65.6	78.2	0.85
SI Contrast	80.4	75.0	78.2	0.89
SI Correlation	82.6	75.0	79.5	0.94
SI Homogeneity	82.6	81.3	82.1	0.93
SI Energy	87.0	65.6	78.2	0.94
SAS	87.0	78.1	83.3	0.95
SAS Contrast	82.6	68.8	76.9	0.89
SAS Correlation	87.0	78.1	83.3	0.93
SAS Homogeneity	80.4	71.9	76.9	0.88
SAS Energy	87.0	59.4	75.6	0.88
ESD	89.1	59.4	76.9	0.84
ESD Contrast	87.0	68.8	79.5	0.92
ESD Correlation	78.3	75.0	76.9	0.88
ESD Homogeneity	87.0	71.9	80.8	0.90
ESD Energy	89.1	65.6	79.5	0.92
EAC	82.6	78.1	80.8	0.90
EAC Contrast	87.0	59.4	75.6	0.88
EAC Correlation	82.6	71.9	78.2	0.95
EAC Homogeneity	93.5	65.6	82.1	0.94
EAC Energy	84.8	78.1	82.1	0.94
Hybrid Vector	95.7	84.4	91.0	0.97

## Discussion and Conclusions

This study demonstrated, for the first time, the potential of QUS spectral and textural analysis techniques for characterization of benign versus malignant breast lesions non-invasively. Ultrasound B-mode images and RF data at clinically-relevant conventional frequencies were collected from the breast lesions of 78 patients. Several QUS parametric maps were generated using spectral analysis techniques in conjunction with a sliding window analysis. Average-based mean-value parameters were determined from each parametric map in addition to four textural features to quantify intra-lesion heterogeneity in tissue micro-structures. The QUS-based biomarkers derived were applied to differentiate between the benign versus malignant lesions non-invasively. The ground truth lesion characteristics were identified from histopathology on biopsy specimens and radiology reports related to clinical MR images. Several QUS biomarkers (one mean-value and 14 textural parameters) demonstrated statistically significant differences between the benign versus malignant lesions. Using a K-NN classifier with leave-one-patient-out cross-validation, several single biomarkers including two mean-value and six textural parameters could classify the lesions with a greater than 80% accuracy. A hybrid vector of biomarkers developed using the stepwise feature selection method achieved a sensitivity of 96%, a specificity of 84%, and an AUC of 0.97.

Among the mean-value parameters, only SAS demonstrated a statistically significant difference between the two lesion types. This can be due to the fact that the average size of lesions in this study was relatively large (2.2 ± 1.7 cm) and QUS parametric images frequently demonstrated considerable levels of spatial heterogeneity within tumour area. Average-based parameters characterize a lesion by only a mean value and do not carry any information regarding intra-lesion heterogeneity. Due to the high level of spatial variations observed in parameter values within lesions, the average of parameters over the entire lesion may not represent its micro-structure appropriately. Therefore one can expect that mean-value parameters cannot differentiate between the two lesion types effectively. This is in agreement with the observations of another study that applied QUS spectral analysis techniques to differentiate between different grades of locally advanced breast cancer^3^. In that study, similarly no QUS mean-value parameter except SAS demonstrated any statistically significant difference between the tumour grades.

The QUS textural parameters determined here demonstrated better performance in differentiating between benign versus malignant lesions. Particularly, among the textural parameters six single biomarkers showed statistically significant differences (p < 0.05), six demonstrated statistically highly significant differences (p < 0.01), and two exhibited statistically extremely significant (p < 0.001) differences between the two lesion types. QUS textural parameters quantify intra-lesion heterogeneities in size, density and the distribution of acoustic scatterers. Therefore, these parameters can potentially characterize tissue micro-structure from different perspectives and provide a better separation between different histological tissue types compared to mean-value parameters. A number of other studies have applied texture-analysis techniques with different imaging modalities including PET, MRI, CT and ultrasound in various diagnostic and prognostic applications^38–44^. In line with the observations in this study, those studies also reported a favorable potential of using imaging-based texture-analysis techniques to characterize heterogeneity in tumour micro-structure, perfusion, physiology, and cell death with diagnostic and prognostic values for cancer characterization.

Results of stepwise linear discriminant analysis demonstrated that a combination of mean and textural parameters in form of a hybrid QUS biomarker provided a better separation between the two lesion types with “extreme” statistical significance (p < 0.001). Such an observation implies that whereas the textural biomarkers can generally better quantify unique micro-structure of each lesion type, the QUS mean-value parameters can provide near-orthogonal information reflecting major difference in histological characteristics of the lesion types in order to form a robust hybrid biomarker. This is in agreement with findings of previous studies where a combination of QUS mean-value and textural parameters demonstrated a better performance in grading breast tumours as well as in detecting cell death-related alterations in tissue micro-structure^3,22^. Classification results obtained in the study here using a leave-one-patient-out cross-validation scheme also suggested a higher potential of QUS textural biomarkers compared to mean-value parameters for breast-lesion categorization. Similarly, a combination of textural and mean-value parameters resulted in a higher sensitivity and specificity for breast-lesion classification. The number of cases in this study (n = 78) is more than 10 times greater than the number of parameters in the hybrid biomarker (n = 7) applied for classification. However further studies on larger cohorts of patients are required to ensure that the classification results are repeatable and not affected by possible excessive dimensionality of the feature vector.

A recent study reported good potential for tumour-core-to-margin ratio of QUS parameters to characterize breast tumour aggressiveness and predict its response to chemotherapy^56^. Benign and malignant lesions of breast often demonstrate different marginal characteristics^64^. Therefore, the QUS core-to-margin ratios may potentially improve the performance of the lesion characterization framework proposed in this study. Such approach has been planned to be investigated in a future study on larger cohort of patients.

Other imaging modalities including x-ray mammography, standard ultrasound (B-mode), and contrast-enhanced MRI are conventionally applied in clinic for breast cancer diagnosis. Compared to these imaging modalities, QUS techniques do not use ionizing radiation for imaging, and can provide quantitative measures that are independent of instrument specifications and scan-session parameters for objective tissue characterization. Also QUS methods do not require injection of any exogenous contrast agents since they rely on the physical and acoustic properties of tissues as source of imaging contrast.

In summary, the rapid characterization of breast lesions is an important component of breast cancer diagnosis that can keep more therapeutic options available for patients and improve survival and quality of life. In this context, non-invasive methods such as QUS texture-analysis framework proposed in this study can facilitate early characterization of breast lesions by providing complementary information on heterogeneous micro-structure of tissue. The results obtained in this study demonstrated a high potential for textural characteristics of QUS parametric maps to be applied in rapid diagnosis of cancerous breast tumours. This work provides a basis for future clinical studies in which the described framework is evaluated on larger cohorts of patients to assess its capabilities further for robust, non-invasive and accurate characterization of benign versus malignant breast lesions.
